# K_ca_3.1 Activation Via P2y_2_ Purinergic Receptors Promotes Human Ovarian Cancer Cell (Skov-3) Migration

**DOI:** 10.1038/s41598-017-04292-6

**Published:** 2017-06-28

**Authors:** L. Robles-Martínez, E. Garay, M. G. Martel-Gallegos, A. Cisneros-Mejorado, D. Pérez-Montiel, A. Lara, R. O. Arellano

**Affiliations:** 1 Departamento de Neurobiología Celular y Molecular, Instituto de Neurobiología, Universidad Nacional Autónoma de México, Boulevard Juriquilla 3001, Juriquilla Querétaro, CP 76230 Querétaro, México Mexico; 2 Departamento de Patología, Instituto Nacional de Cancerología, Secretaría de Salud, Av. San Fernando #22, Colonia Sección XVI, Tlalpan, CP 14080 Ciudad de México, México Mexico

## Abstract

Disorders in cell signaling mediated by ATP or histamine, activating specific membrane receptors, have been frequently associated with tumorigenesis. Among the elements of response to purinergic (and histaminergic) signaling, ion channel activation controls essential cellular processes in cancer, such as cell proliferation, motility, and death. Here, we studied the effects that ATP had on electrical properties of human ovarian adenocarcinoma cells named SKOV-3. ATP caused increase in intracellular Ca^2+^ concentration ([Ca^2+^]_i_) and, concurrently, it evoked a complex electrical response with a conspicuous outward component. This current was generated through P2Y_2_ receptor activation and opening of K^+^ channels, K_Ca_3.1, as indicated by electrophysiological and pharmacological analysis, as well as by immunodetection and specific silencing of P2Y_2_ or K_Ca_3.1 gene by esiRNA transfection. Low µM ATP concentration increased SKOV-3 cell migration, which was strongly inhibited by K_Ca_3.1 channel blockers and by esiRNA-generated P2Y_2_ or K_Ca_3.1 downregulation. Finally, in human ovarian tumors, the P2Y_2_ and K_Ca_3.1 proteins are expressed and co-localized in neoplastic cells. Thus, stimulation of P2Y_2_ receptors expressed in SKOV-3 cells promotes motility through K_Ca_3.1 activation. Since P2Y_2_ and K_Ca_3.1 are co-expressed in primary tumors, our findings suggest that they may play a role in cancer progression.

## Introduction

Evidence supports a relationship between alterations in the purinergic or histaminergic signaling systems and the cancer process in several cell types^1, 2^. Thus, stimulation of specific, ATP-sensitive membrane receptors, named P2 receptors, inhibits cell growth and/or promotes apoptosis in various cancer cells such as breast cancer^3^, cervical cancer^4^, glioma^5^, and prostate cancer^6^, among many others. However, purinergic stimulation might also have the opposite effect as it can promote cell proliferation, either in distinct cancer cell types or even in the same model when tested in different experimental conditions. These divergent effects have been thought to reflect ATP availability in the tumor environment together with a specific combination of purinergic membrane receptors expressed in a particular cell type^1^, and in addition, they would be strongly influenced by the expression of a distinctive set of effector proteins, such as G proteins, protein kinases, and membrane ion channels. Histaminergic signaling that is altered in cancer has also been proposed as an important paracrine and autocrine regulator of proliferation^2^, as well as a mediator of cancer progression acting on cell migration, angiogenesis, and modulation of the immune response.

Previous studies indicated that ion channel function might be one of the modifications suffered in cancer; their activation or inhibition, for example, affects various important functional processes in the context of cancer^7–10^. Altered expression of a diversity of K^+^ channels in human breast cancer cells, in human astrocytomas and glioblastomas, and in human ovarian cells including SKOV-3 have been documented in distinct cell models^11, 12^. Although ion channel activation through purinergic receptor stimulation is a well-known phenomenon, its role in cancer has not been thoroughly analyzed. Here, we undertake an analysis of the effects mediated by ATP (and histamine) on the electrical properties of human ovarian cancer cells named SKOV-3^13^, a well-studied cell model that expresses molecular markers of epithelial to mesenchymal transition, a phenomenon associated with tumor metastasis^14^.

SKOV-3 cells are endowed with P2 receptors of the two known subtypes: those forming receptor-channels named P2X^15^, as well as G protein-coupled receptors named P2Y. ATP application generates in SKOV-3 an increase in the intracellular Ca^2+^ concentration ([Ca^2+^]_i_) via its release through P2 receptor stimulation^16^, and a similar [Ca^2+^]_i_ increase is evoked by histaminergic signaling activation; the effect of this [Ca^2+^]_i_ increase on membrane conductance, however, remains to be explored. On the other hand, the expression and function of K^+^ channels correlate with the cancer progression in SKOV-3 cells, as some specific K^+^-channel subtypes, such as two-pore K^+^ channels, are upregulated^17, 18^.

Here, we carried out electrophysiological studies of SKOV-3 cells stimulated by ATP and other drugs, and found that specific stimulation of P2Y_2_ receptors generated mainly an outward current response carried by K^+^ and that this was mimicked by histamine. We also showed that the K_Ca_3.1 channel activation was a prompt, electrical response to ATP or histamine and that it promoted SKOV-3 cell migration, while specific silencing of K_Ca_3.1 or P2Y_2_ gene downregulated protein expression and strongly reduced both the electrical response and cell motility. Finally, we provide evidence that both K_Ca_3.1 channels and P2Y_2_ receptors are expressed in SKOV-3 cells and in neoplastic cells in human ovarian tumor biopsies. Thus, we propose that K_Ca_3.1 channels are important for the tumorigenic process, specifically by promoting cellular migration. This information suggests that K_Ca_3.1 channels might be a useful target for the development of diagnostic and therapeutic strategies against ovarian cancer.

## Results

### ATP triggers complex electrical membrane responses in SKOV-3 cells

**Figure 1:**
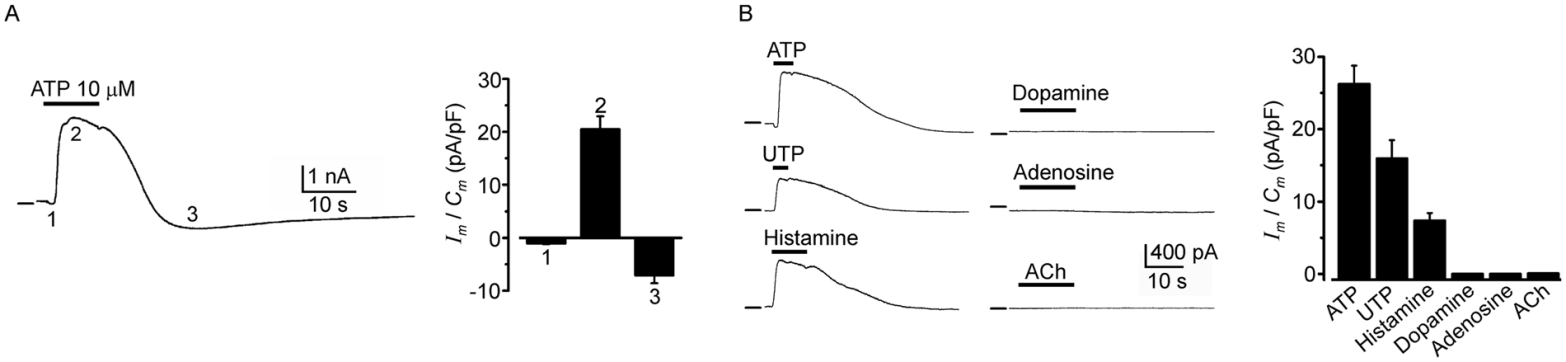
SKOV-3 cells respond to ATP by generating multiphasic ionic currents. (**A**) The trace shows a typical electrical response generated in SKOV-3 cells by ATP application; in cells held at −40 mV, three main components are identified in the response: 1) a fast inward current with low amplitude, 2) a robust outward current, and 3) a slow inward current. All response components were associated with an increase in membrane conductance. The bar graph shows the current density recorded for each component in a sample of 39 cells. (**B**) Traces show that component 2 was also activated by UTP or histamine application. All agonists were applied at 10 µM, and the bar graph summarizes the outward current density recorded in 60 cells tested for the various agonists. In general, in this and subsequent traces, responses were obtained in cells held at −40 mV, and upper bars indicate the drug application time in each case. Horizontal lines indicate zero current.

Electrophysiological experiments were made in SKOV-3 cells between passages 1 to 6, within a period of 48–72 h in culture. Single cells, or those with no more than 1 or 2 in contact, were chosen in order to avoid extensive cell coupling, although it has been shown previously that SKOV-3 cells have a low level of cell-to-cell coupling mediated through gap junction channels under similar culture conditions^19^. Thus, SKOV-3 cells presented a C_m_ of 48.9 ± 1.5 pF and R_m_ of 127.5 ± 17.8 MΩ (359 cells) as monitored in whole cell configuration using the standard internal and external solutions. SKOV-3 cells held at −40 mV a value close to their resting membrane potential (−38.8 ± 2.4 mV) were systematically tested for different agonists. ATP (1–100 µM) application elicited in 92.3 ± 3% of the cells a complex electrical response consistently composed of 3 main components (denoted components 1–3 in Fig. 1A), which was associated with an increase in membrane conductance: a fast, inward response of low amplitude (1) followed by a robust outward current (2) that developed together with a smaller, slower, and relatively less frequent inward current (3). Component 2 was the main membrane current elicited by ATP, and it had an average amplitude of 26.24 ± 2.5 pA/pF (n = 186 cells). Typically, the current response to ATP started to decrease after 2 to 3 applications with washing intervals of 3–4 min, and the response disappeared completely after 5 to 8 sequential applications (data not shown).

Other agonists were tested for their ability to activate membrane currents (Fig. 1B). Among them, UTP (n = 33) or histamine (n = 9) also generated current responses that mimicked mainly component 2 (12.91 ± 2.1 pA/pF and 7.3 ± 1.05 pA/pF; for UTP and histamine, respectively). It appears that current component 1 is specific for ATP. All other agonists, such as acetylcholine, adenosine or dopamine (Fig. 1B), failed to activate any response (4 cells in each case). Thus, these data strongly suggest that current responses were specific for purinergic and histaminergic receptors. We also explored the ATP sensitivity of the ovarian adenocarcinoma cell line named CaOV-3 (n = 10), where 10 µM ATP application generated similar current components 1 and 2, with an average amplitude of 0.47 ± 0.007 pA/pF and 17.4 ± 3.8 pA/pF, respectively.

### [Ca^2+^]_i_ increase elicited by agonists, and dose-response relationships for outward electrical response

**Figure 2:**
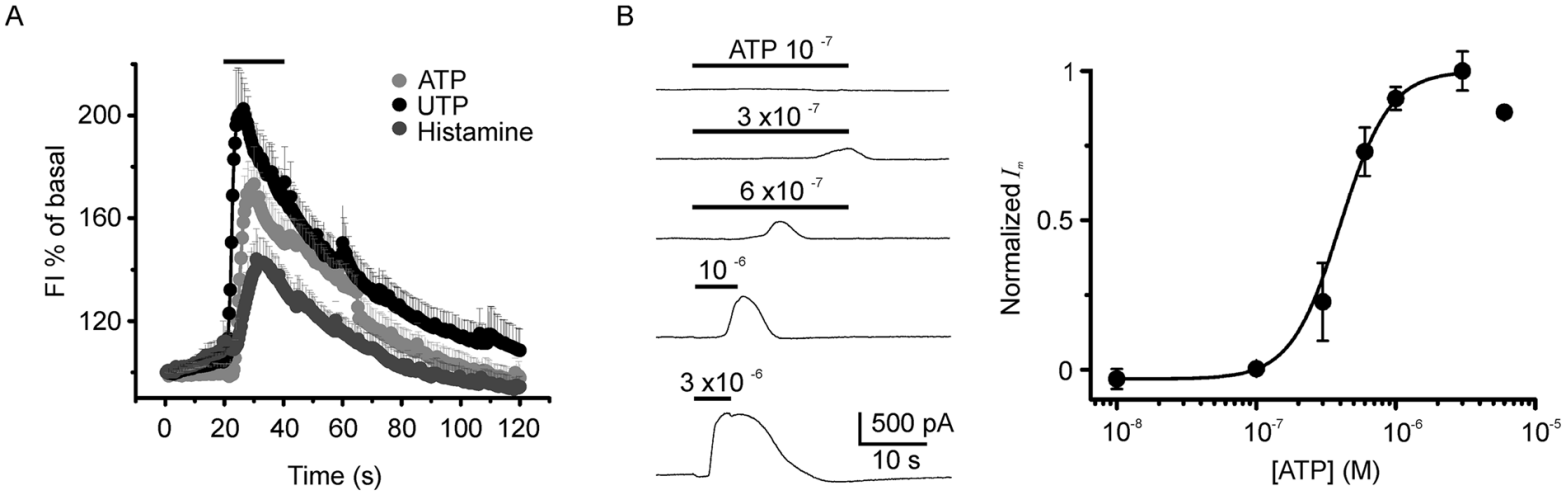
Intracellular Ca^2+^ concentration increase in SKOV-3 cells and dose-response of the current generated by ATP. (**A**) Agonists that generated an electrical response also elicited a [Ca^2+^]_i_ increase that was monitored fluorometrically in cells loaded with Fluo4-AM. All three agonists (10 µM) elicited a fast response in most cells studied; the graph shows the increase in fluorescence intensity as a percentage of the basal level in 88 cells from different cell cultures. (**B**) Traces correspond to current responses elicited in the same cell that was stimulated with increasing ATP concentrations (washing intervals of 5 min between each application). Similar experiments were repeated in 20 cells, and the peak outward current for each ATP concentration was normalized against the maximal response (at 3 µM ATP), averaged and plotted. Curve is the fit to the equation: I/Imax = [(A1 − A2)/1 + ([ATP]/EC_50_)^nH^] + A2; by the method of non-linear least squares fitting, where EC_50_ = 399 ± 11.4 nM is the half-maximal effective concentration of ATP, nH = 2.45 ± 0.06 is the Hill coefficient, and A1 and A2 are the initial and final normalized I-values, respectively.

We next examined whether ATP or histamine application in SKOV-3 cells produced a [Ca^2+^]_i_ increase using fluorometry. As shown in Fig. 2A, P2Y stimulation by 10 µM ATP or UTP generated a robust Ca^2+^ increase that was mimicked by 10 µM histamine, as previously shown^16^.

As illustrated in Fig. 2B, it was also demonstrated that the electrical response generated by ATP application was dose dependent; in these experiments, cells held at -40 mV were perfused with increasing concentrations of ATP within the range of 0.1 to 30 µM with wash intervals of 4–5 min. The peak response at each concentration was normalized against the maximal current that occurred around 3 µM. The dose-response curve gave an estimated EC_50_ of 399 ± 11.4 nM for component 2 elicited by ATP.

### Outward membrane response to ATP was mimicked by purinergic agonists

**Figure 3:**
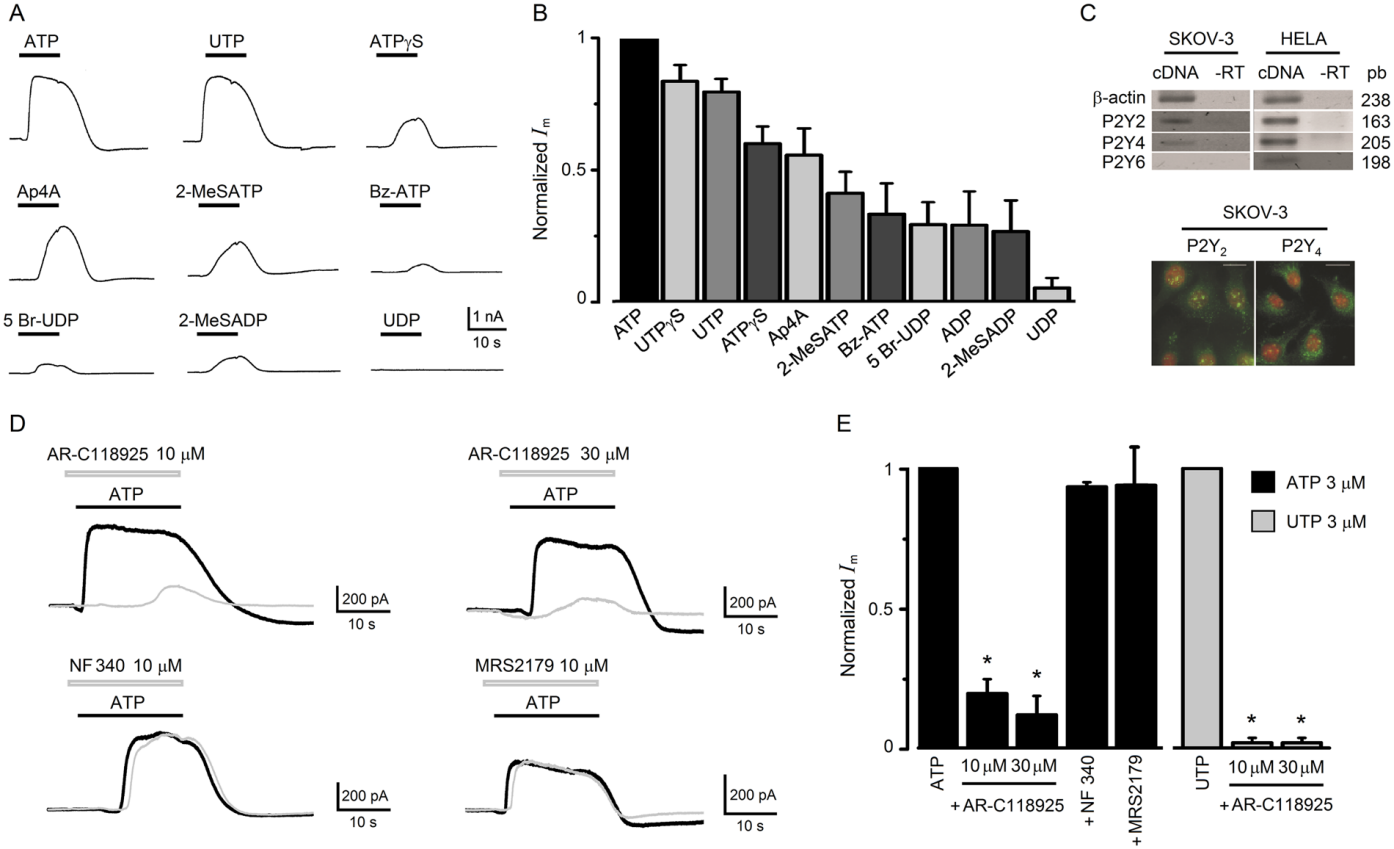
Purinergic pharmacology and expression of the receptor involved in the electrical response in SKOV-3 cells. (**A**) A battery of different agonists (all applied at 3 µM) for purinergic receptors was tested in cells held at −40 mV, and the peak current amplitude for each agonist was normalized against that obtained by applying ATP. This resulted in the potency sequence illustrated in graph (**B**) where data obtained in 50 cells are summarized. (**C**) The upper panel shows the PCR analysis for P2Y receptors sensitive to ATP/UTP; P2Y_2_, P2Y_4_ and P2Y_6_ receptor subtypes. In SKOV-3 cells, bands with the expected size were obtained for P2Y_2_ and P2Y_4_ subtypes but not for P2Y_6_ (mRNA from HELA cells was used as positive control, and -RT indicates the corresponding reaction control without reverse transcriptase). The images show immunorecognition for P2Y_2_ or P2Y_4_ receptor proteins in SKOV-3 cells observed by epifluorescence (P2Y receptor in green and nuclei in red labeled with propidium iodide). Scale bar 10 µm. (**D**) Traces illustrate the effect of AR-C118925, NF340, or MRS2179, antagonists of P2Y_2_, P2Y_1_ or P2Y_11_ receptor, respectively. Each set of records corresponds to the current response elicited by ATP (3 µM; black trace) alone or in the presence of an antagonist (gray trace) as indicated. (**E**) The graph summarizes the results obtained in experiments as in (**D**). Each bar corresponds to the current amplitude in every condition (n = 16) normalized against the current obtained by applying ATP alone. The graph also shows results of using UTP as agonist and AR-C118925 as antagonist (n = 16; *p < 0.05).

In order to distinguish among different P2 receptors, a battery of agonists was tested, all at 3 µM concentration (Fig. 3A). In Fig. 3B the bars illustrate the current response elicited by the different drugs, normalized against that obtained by ATP application in the same cell. This gave the following sequence of potency: ATP > UTPγS > UTP > ATPγS ≥ Ap4A > 2-MeSATP ≫ Bz-ATP ≥ 5 Br-UDP ≥ ADP ≥ 2-MeSADP ≫> UDP; with no response to adenosine. All agonists tested generated mainly the outward current, although the more potent drugs, such as UTP, also elicited component 3 in a way similar to ATP, but not component 1. This strongly suggested that the receptor involved in generating the outward current was one of the P2Y subtypes sensitive to UTP with a pharmacological profile close to that displayed for the P2Y_2_ subtype.

Three different P2Y-type receptors sensitive to UTP are known in different species^20, 21^; these are named the P2Y_2_, P2Y_4_ and P2Y_6_ receptors. Thus, an RT-PCR analysis was made to specifically amplify the transcripts for these receptor subtypes in SKOV-3 cells, and it showed that P2Y_2_ and P2Y_4_ were well expressed while P2Y_6_ was not amplified (Fig. 3C). Immunocytochemistry against these receptors in SKOV-3 cells indicated that P2Y_2_ and P2Y_4_ proteins were also well expressed in 90% of cells tested (Fig. 3C). However, it is well known that the human P2Y_4_ receptor is not activated by ATP^22^. All these data strongly suggested a main role of P2Y_2_ in ATP- or UTP-elicited outward current response of SKOV-3 cells. This was also supported by the inhibitory effect of AR-C118925 (10–30 µM), an antagonist with high specificity for P2Y_2_ receptors^23^, that reduced 80–88% of the response elicited by ATP (3 µM) and more than 98% of the response to UTP (3 µM) (Fig. 3D,E); while NF340 or MRS2179, specific antagonists for P2Y_11_ and P2Y_1_ subtypes^21^, respectively, did not affect SKOV-3 responses generated by ATP.

### Ionic basis for the outward current generated by ATP

**Figure 4:**
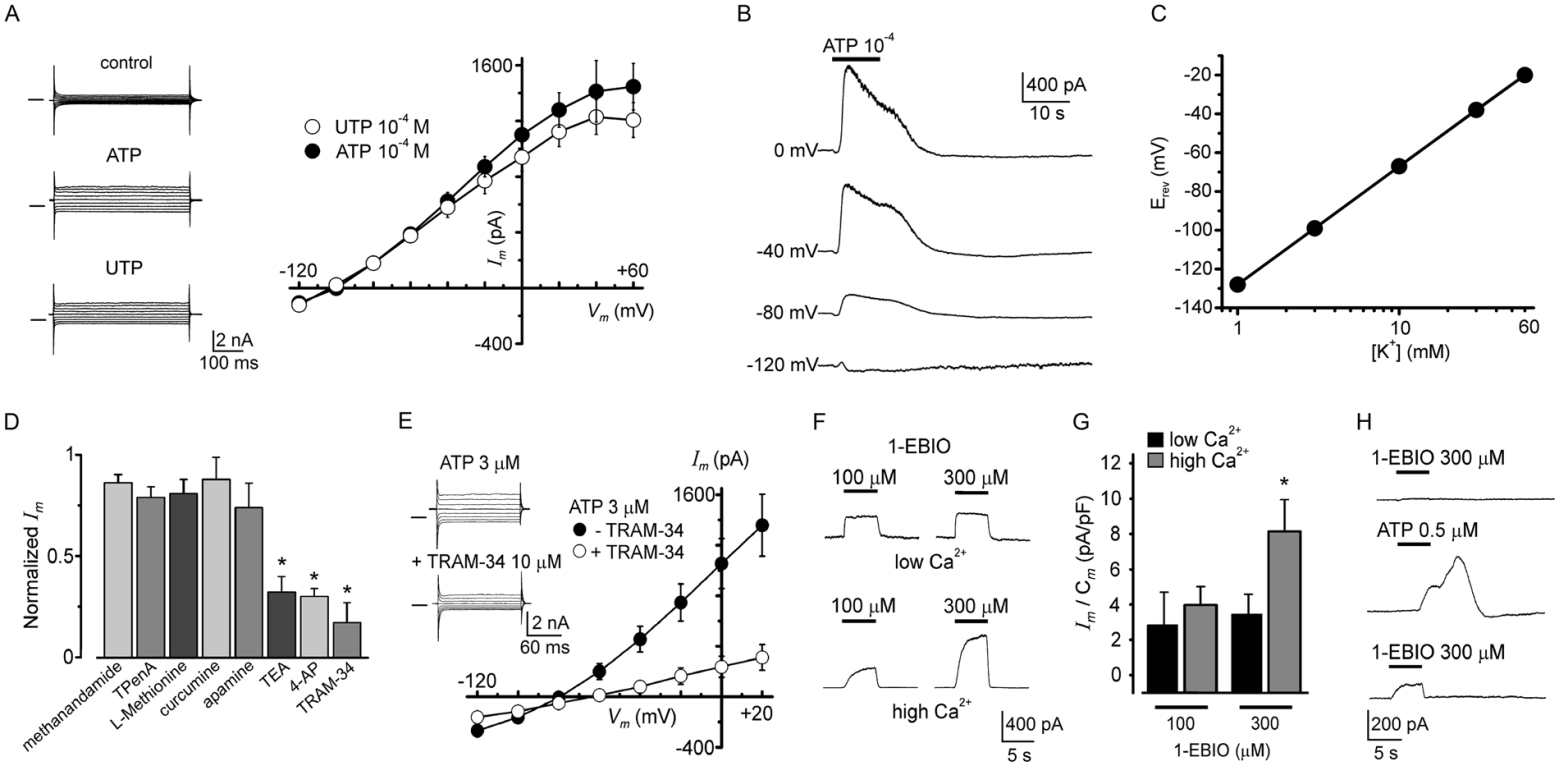
Ionic basis of the outward current elicited by ATP and effect of K^+^ channel modulators. (**A**) Current responses obtained by applying voltage pulses, from −120 to +60 mV in 20 mV steps, to a SKOV-3 cell held at −40 mV. Currents during superfusion in control conditions (upper traces) and at the outward component peak elicited either by ATP (middle traces) or UTP (lower traces). Control currents were subtracted from those obtained at the peak response, and the amplitude values were plotted against the respective membrane voltage; the linear I/V relationship obtained (23 cells) showed a reversal membrane potential close to −100 mV. (**B**) Traces illustrate an example of current responses elicited by ATP while a cell was held at different values as indicated, confirming a linear I/V relationship and the E_rev_ estimation for the outward component. (**C**) Relationship between E_rev_ (estimated as in (**A**) and external K^+^ concentration; line is the Nernst relationship considering internal and external concentrations in each case. Data represent means ± SEM of 10–15 cells. (**D**) A battery for distinct K^+^-channel inhibitors was tested on the response, including non-specific blockers of K^+^ channels (TEA^+^ and 4-AP), those specific for two-pore K^+^ channels, and blockers apamin and TRAM-34 specific for K_Ca_2 and K_Ca_3.1, respectively; bars indicate the proportion of current inhibited by the different antagonists (*p < 0.05). (**E**) Traces show membrane current recorded during the outward peak response to ATP (upper traces) and that obtained in the same cell by co-applying ATP together with TRAM-34 (10 µM), a specific blocker of K_Ca_3.1 channels (lower traces). The graph shows data (means ± SEM) obtained in 30 cells under the same protocol in which the basal currents were subtracted for each case, averaged and plotted. (**F**) Currents elicited by 1-EBIO (100 or 300 µM), a K^+^ channel opener specific for K_Ca_3.1 channels, applied at two different intracellular Ca^2+^ concentrations: low and high (estimated concentration of 10 and 300 nM, respectively). The bar graph shows the averaged current density obtained in each condition monitored in 14 cells. (**G**) Traces illustrate a case in which initially, 1-EBIO was unable to generate any current (upper trace); however, after a response elicited by ATP, a second application of 1-EBIO (lower trace) at the same concentration generated a meaningful outward response.

Current-voltage (I/V) relationships were built by applying voltage steps in control conditions and at the peak of the outward current activated by ATP; the differences between these two relations were plotted as illustrated in Fig. 4A. The I/V curve showed that the outward current activated had a reversal potential (E_rev_) of –95 ± 5 mV (n = 54), which corresponded closely to that for K^+^ ions in the recording conditions. Similar voltage dependency was also observed in experiments where cells were held at different potentials and then tested by applying 3 µM ATP. As shown in Fig. 4B, component 2 of the response inverted polarity close to −100 mV. Using the voltage-stepping protocol and increasing the concentration of K^+^ (substituted for Na^+^) in the external solution, the current E_rev_ shifted to less negative values; thus in 60 mM K^+^ it was −20 ± 9 mV, and in 30 mM K^+^ the E_rev_ was −35 ± 5 mV. As shown in Fig. 4C the logarithmic relation of E_rev_ versus extracellular K^+^ concentration was a line with a slope of −59 mV, in agreement with the Nernst equation. Also, I/V curves were built during the development of component 3 after the complete wash out of component 2; in this condition inward currents had an E_rev_ of −2 ± 1 mV (n = 10) and were not affected by T16Ainh-A01 (100 µM; n = 5), a specific Ca^2+^-dependent Cl^−^ channel blocker.

### Effect of K^+^ channel blockers and expression of K_Ca_3.1 channels

**Figure 5:**
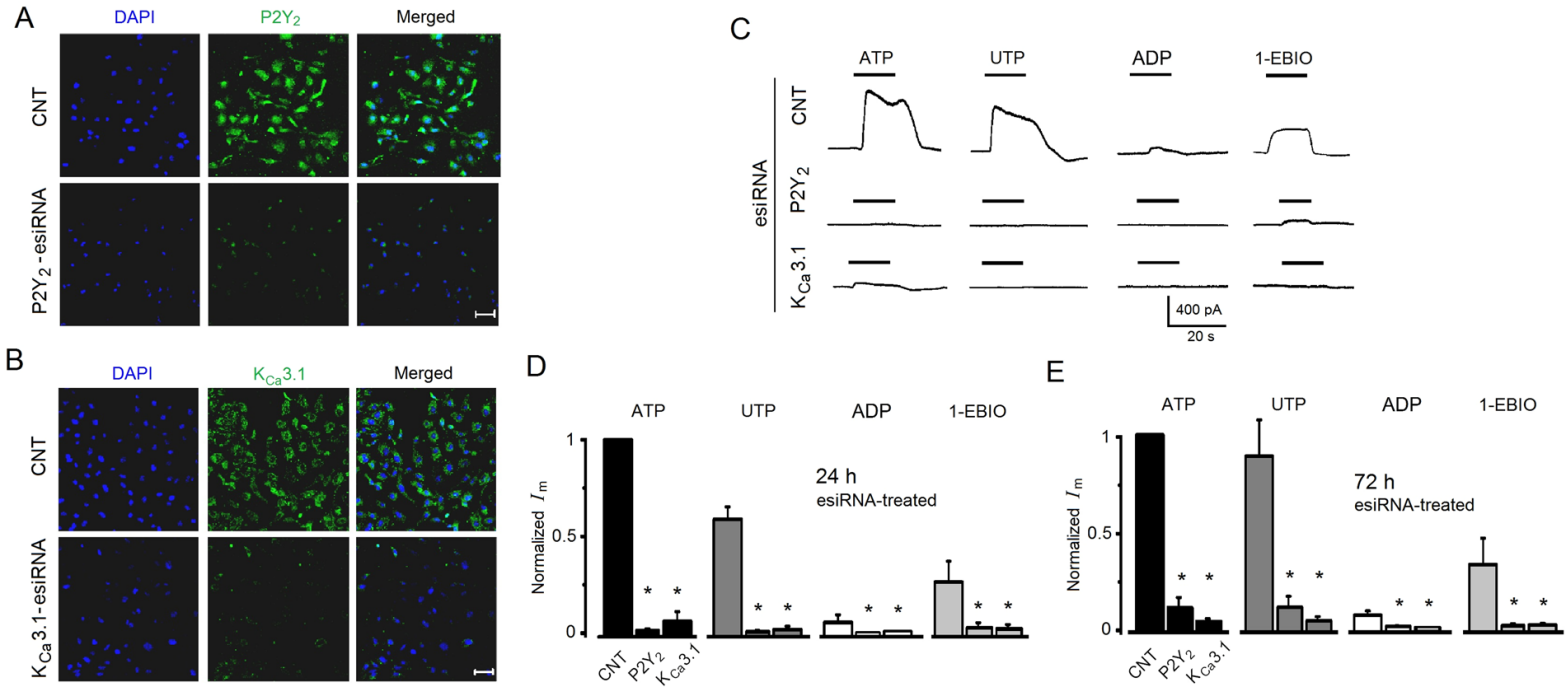
P2Y_2_ and K_Ca_3.1 protein expression and electric response to agonists in esiRNA transfected SKOV-3 cells. (**A**) Analysis by immunocytochemistry after 48 h of esiRNA treatment in control (CNT) and P2Y_2_-esiRNA-treated groups. Panels show the fluorescence signal for DAPI (blue) in the first column, the signal obtained with a specific antibody against P2Y_2_ receptor protein (in green) in the second column, and the corresponding merged image. (**B**) Similar analysis was made in K_Ca_3.1-esiRNA-treated cells using an antibody against K_Ca_3.1 channel protein (in **A** and **B** bar = 50 µm). (**C**) Traces illustrate current response elicited by agonists (all 3 µM) or 1-EBIO (300 µM) in SKOV-3 cells 24 h after esiRNA transfection. In (**D** and **E**) traces illustrate current responses in SKOV-3 cells 24 h and 72 h after esiRNA transfection, respectively. Currents were normalized against the ATP-elicited response in the CNT group (18–19 cells in each condition). Every set of bars corresponds to either CNT, P2Y_2_-esiRNA, or K_Ca_3.1-esiRNA as indicated in the group for ATP application (*p < 0.05, experimental condition vs. respective CNT).

Consistent with a conducting pathway for K^+^ ions during component 2, the ATP response was inhibited by 5 mM tetraethylammonium (TEA^+^) or 5 mM 4-aminopyridine (4-AP), unselective K^+^ channel blockers (18 cells for each blocker), by 67 ± 7% and 70 ± 4%, respectively (Fig. 5B). Similar results were obtained for outward currents activated by UTP. Together, these results indicated that purinergic activation opened a conductance selective for K^+^ ions; thus, we tested various drugs that act selectively on K^+^ channels that have been shown to either be expressed in SKOV-3 cells or be involved in tumor biology (Fig. 4D–H). Specific blockers of two-pore K^+^ channels known to be overexpressed in SKOV-3 cells^17, 18^, such as 20 µM curcumin, 100 µM L-methionine, 100 µM TPenA (a TREK-1 and TREK-2 blocker), and 20 µM methanandamide (a TASK-3 blocker), showed only small inhibitory effects (ranging from 12 to 26%) on the K^+^ current elicited by ATP (or UTP; n = 22) that were not significant when compared with the amplitude of control currents (Fig. 4D). However, application of 10 µM TRAM-34, a specific blocker for intermediate-conductance Ca^2+^-dependent K^+^ channels^24^ (K_Ca_3.1), produced a strong inhibition of 83 ± 9% (n = 12) of the current response elicited by ATP or UTP (Fig. 4D,E), while 1 µM apamin, a potent blocker of the small-conductance Ca^2+^-dependent K^+^ channel (K_Ca_2) subtype, had a smaller inhibitory effect of 26 ± 10% (n = 12) that was not significant (Fig. 4D). Accordingly, the drug 1-EBIO (100–300 µM), a specific opener for K_Ca_3.1 channels, produced the generation of outward currents in SKOV-3 cells in a manner that was dependent on drug concentration as well as on the free Ca^2+^ concentration in the internal solution (Fig. 4F–H)^25–27^. Also, it was noted that in cells where initial application of 300 µM 1-EBIO was not able to generate a response (6 out of 18 cells), pre-stimulation with a low dose of 0.5 µM ATP (256.6 ± 61 pA) primed the cells to respond to a second application of 1-EBIO (396 ± 66.7 pA; Fig. 4H). These results indicated that purinergic stimulation of P2Y receptors elicited the activation of K^+^ channels sensitive to TRAM-34 and 1-EBIO, which are specific drugs for K_Ca_3.1 channels.

**Figure 6:**
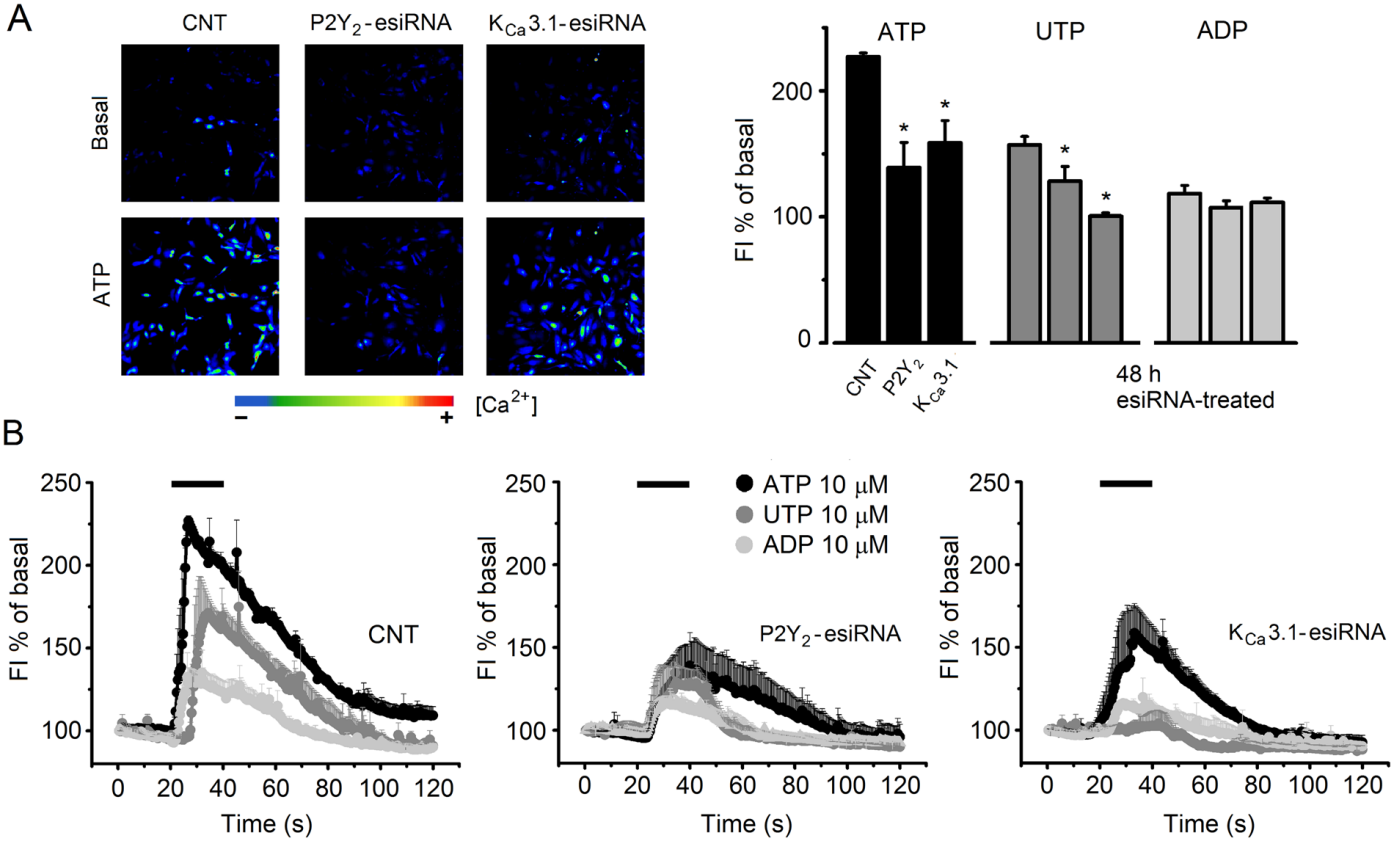
Intracellular Ca^2+^ concentration increase in esiRNA-treated SKOV-3 cells. (**A**) Images illustrate the [Ca^2+^]_i_ increase elicited by ATP (10 µM) monitored fluorometrically in SKOV-3 cells loaded with Fluo4-AM. Cells were esiRNA treated 48 h before the experiment. The first row illustrates basal fluorescence a few seconds before ATP application, and second row images in the peak of the response. The graph summarizes the peak fluorescence intensity change generated either by ATP, UTP or ADP (all 10 µM), as a percentage of the basal level, for cells in every group as indicated in the ATP set (*p < 0.05, experimental condition vs. respective CNT). (**B**) Time-course of the fluorescence intensity change generated either by ATP, UTP or ADP in each group of esiRNA-treated cells (after 48 h). Every point corresponds to the average fluorescence intensity ± S.E.M. of 331–690 cells from 2 different cultures.

A Western blotting was performed to confirm K_Ca_3.1 protein expression in SKOV-3 cells. The results are illustrated in Supplementary Figure S1A where it is shown that an antibody specific against this channel subtype gave a band with the expected weight of 55 KDa for the K_Ca_3.1 channel protein. Immunocytochemical analysis of SKOV-3 cells also showed strong, specific label in most cells in the culture; a positive control using neurons from cortex^28^ is shown in Supplementary Figure S1B (see also Fig. 6A and Supplementary Figure S2).

### P2Y_2_ and K_Ca_3.1 gene silencing by specific esiRNA transfection

Functional and pharmacological results suggested that the purinergic receptor and the ion channel involved in K^+^ current response were P2Y_2_ and K_Ca_3.1, respectively. Thus, knockdown of either P2Y_2_ or K_Ca_3.1 protein was made silencing the respective gene, transfecting SKOV-3 cells with specific esiRNA (Fig. 5); then effects of silencing were tested monitoring the electrical response or [Ca^2+^]_i_ change elicited by agonists. Responses were compared with those of cells transfected in parallel with esiRNA of enhanced green fluorescent protein as control (CNT) group. First, esiRNA-transfected SKOV-3 cells were analyzed (24–72 h after transfection) by immunocytochemistry to detect specific expression of P2Y_2_ or K_Ca_3.1 protein. Images in Fig. 5A and B illustrate the results; in both cases either P2Y_2_-esiRNA or K_Ca_3.1-esiRNA transfected cells showed a strong signal decrease for the respective protein. SKOV-3 cells transfected with P2Y_2_-esiRNA maintained basal expression of K_Ca_3.1 channel protein, while K_Ca_3.1-esiRNA transfected cells kept P2Y_2_ receptor expression (Supplementary Figure S2). esiRNA-transfected SKOV-3 cells, including the control group, were electrophysiologically monitored for responses elicited by ATP or UTP, the more potent agonists; ADP, the weak agonist; and 1-EBIO, the K^+^-channel positive modulator (Fig. 5C). Both groups of esiRNA-transfected cells showed a strong decrease in electrical response elicited by drug application. A pool of 85 cells from 2–3 different transfections (24–72 h) (Fig. 5D,E) showed that current responses remained low at least for 72 h. For example, 24 h after P2Y_2_-esiRNA transfection, ATP response decreased by 90.65 ± 4.7%, UTP by 87.75 ± 6.5%, while ADP and 1-EBIO decreased by 83.15 ± 3.9% and 96 ± 3.1%, respectively. Similar results were obtained in K_Ca_3.1-esiRNA transfected cells. It was consistently observed that knocking down P2Y_2_ receptor also produced a strong decrease in 1-EBIO response (16.92 ± 7.4 pA; n = 19). Nevertheless, as that shown in Fig. 4H, after ATP (3 µM) application, a larger response was activated in P2Y_2_-esiRNA treated cells tested with a second 1-EBIO (300 µM) superfusion, although amplitudes were significantly decreased compared with control responses (96.65 ± 42.4 pA vs. 539.3 ± 75.1 pA; n = 15). This was not observed in K_Ca_3.1-esiRNA transfected cells where a second 1-EBIO application, after ATP, was ineffective (not shown).

SKOV-3 silenced for P2Y_2_ or K_Ca_3.1 protein were fluorometrically monitored for [Ca^2+^]_i_ changes elicited by agonists (ATP, UTP, or ADP) (Fig. 6). The bar graph in Fig. 6A shows that when tested for ATP both groups of esiRNA-transfected cells, P2Y_2_ or K_Ca_3.1, showed a significant reduction of [Ca^2+^]_i_ increase compared with CNT cells. In the case of P2Y_2_-esiRNA, the reduction was of 69.3 ± 2.98% while for K_Ca_3.1-esiRNA it was of 54.3 ± 4%. A similar result was obtained in cells tested with UTP, where [Ca^2+^]_i_ increase was reduced by 51 ± 4% and 98 ± 1.5%, respectively. Thus, downregulation of K_Ca_3.1 alone in SKOV-3 cells unexpectedly seemed to affect [Ca^2+^]_i_ increase elicited by both agonists, suggesting an effect of membrane potential and/or Ca^2+^ influx on the response. In addition, responses elicited by ADP were reduced compared with the control group, although changes were not statistically significant in such cases.

All these results indicated that downregulation of P2Y_2_ or K_Ca_3.1 eliminated the electrical response to ATP or UTP, and that strongly reduced the [Ca^2+^]_i_ increase regularly generated by the same agonists.

### K_Ca_3.1 channel blockage and cell migration elicited by ATP

**Figure 7:**
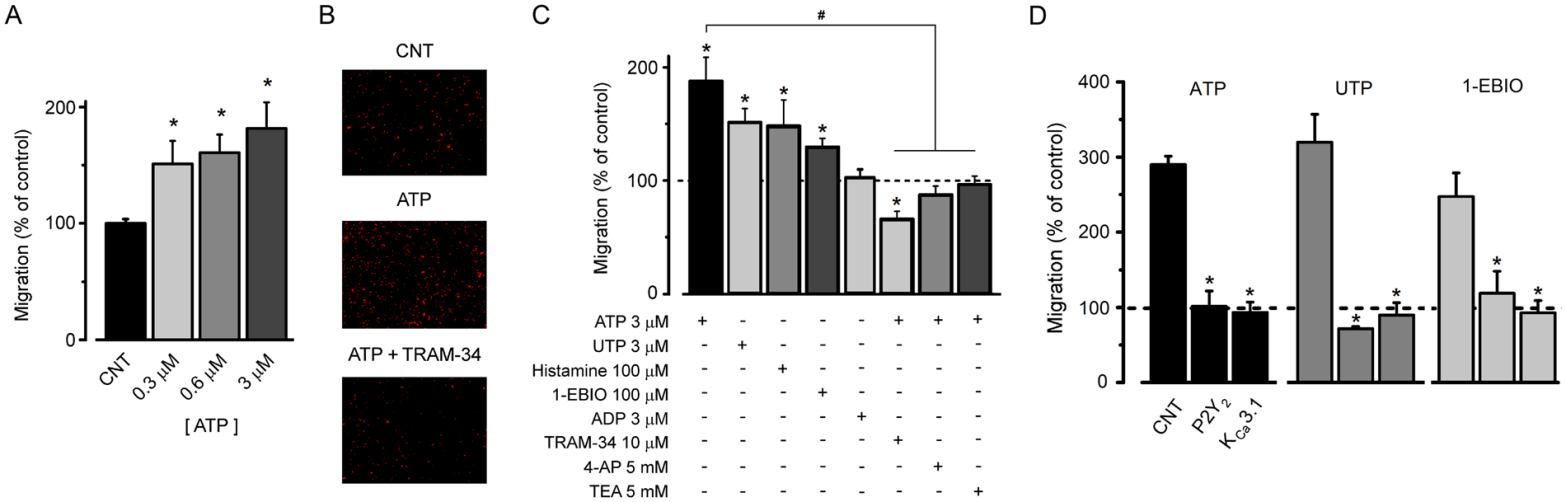
SKOV-3 migration promoted by ATP was inhibited by K^+^ channel blockers or esiRNA treatment. (**A**) Bar graph shows SKOV-3 cell migration quantified after a 16-h incubation with 3 different ATP concentrations (0.3, 0.6, or 3 µM). (**B**) ATP-promoted migration was evaluated in different conditions. Images illustrate cell migration in control condition without added ATP, with 3 µM ATP, and 3 µM ATP co-applied with 10 µM TRAM-34. (**C**) Bar graph shows cell migration normalized against control. Cells were incubated in different conditions as indicated by + signs in experiments like those shown in (**B**) (*p < 0.05, experimental condition vs. control without treatment). Note that drugs that activate the K^+^ response promoted migration, while K_Ca_3.1 blockers (last 3 bars) in the presence of ATP significantly inhibited migration to basal level, or even below, as in TRAM-34 application (*p < 0.05, compared vs. control without treatment). Migration in the presence of K^+^ blockers was also statistically different with respect to ATP-promoted migration (^#^p < 0.05). The data represent means ± S.E.M. of 3 different culture preparations. (**D**) SKOV-3 cells after 48 h of treatment with esiRNA were assayed for migration promoted either by 3 µM ATP, 3 µM UTP or 300 µM 1-EBIO. For each drug treatment the distinct esiRNA transfection conditions were evaluated as in Fig. 5 and indicated in the set stimulated with ATP (*p < 0.05, experimental condition vs. respective CNT).

In several cell types, an important role attributed to K_Ca_3.1 is its involvement in the migration phenomenon^29^ for example in microglia^30^, glioblastoma^31^ as well as in fibroblasts and melanoma cells^32^. First, we asked whether stimulation by ATP promoted cell migration in the concentration range that generated the current response. The results from the transwell migration assay are illustrated in Fig. 7A, where we quantified the migration of SKOV-3 cells for 16 h in the absence or presence of 0.3 µM, 0.6 µM or 3 µM ATP. We observed that migration of cells increased significantly in a dose-dependent manner in the presence of ATP at 0.3, 0.6 and 3 µM to 151.1 ± 19.8%, 160.7 ± 15.6%, and 181.6 ± 22.4%, respectively. Control experiments showed that ATP in the concentrations used did not affect cell proliferation or survival within the 16-h incubation period (using the MTS assay), in agreement with previous studies^16^. Migration of SKOV-3 cells was also stimulated by UTP and histamine, suggesting a close correlation of the effects exerted by receptors that generated a [Ca^2+^]_i_ increase, current generation and migration induction. Thus, we tested whether the K^+^ current carried through K_Ca_3.1 channels was also involved in the ATP-mediated migration increase. This is illustrated in Fig. 7C where it is shown that 100 µM 1-EBIO applied alone increased the phenomenon, while K^+^ channel blockers applied together with ATP, such as TEA^+^, 4-AP or the specific blocker TRAM-34, inhibited cell migration compared to that of ATP alone. Moreover, the former blocker decreased migration with respect to basal level. Similar to the lack of ATP effect on proliferation and cell survival, TRAM-34 alone had no effect in control experiments with SKOV-3.

SKOV-3 cells that were esiRNA-treated were also tested for motility using a similar protocol; the results are illustrated in Fig. 7D. Groups of esiRNA-transfected cells were tested for migration with 3 µM ATP, 3 µM UTP or 300 µM 1-EBIO. The results showed that in P2Y_2_- or K_Ca_3.1-esiRNA transfected cells, cell migration was strongly reduced by all drugs tested (compared with CNT group), including the group of P2Y_2_-esiRNA transfected cells tested with 1-EBIO. This suggested that P2Y_2_ receptor was required for complete channel activation, a notion that was in agreement with our electrophysiological results.

### K_Ca_3.1 and P2Y_2_ receptor expression in biopsies of human ovarian tumors

**Figure 8:**
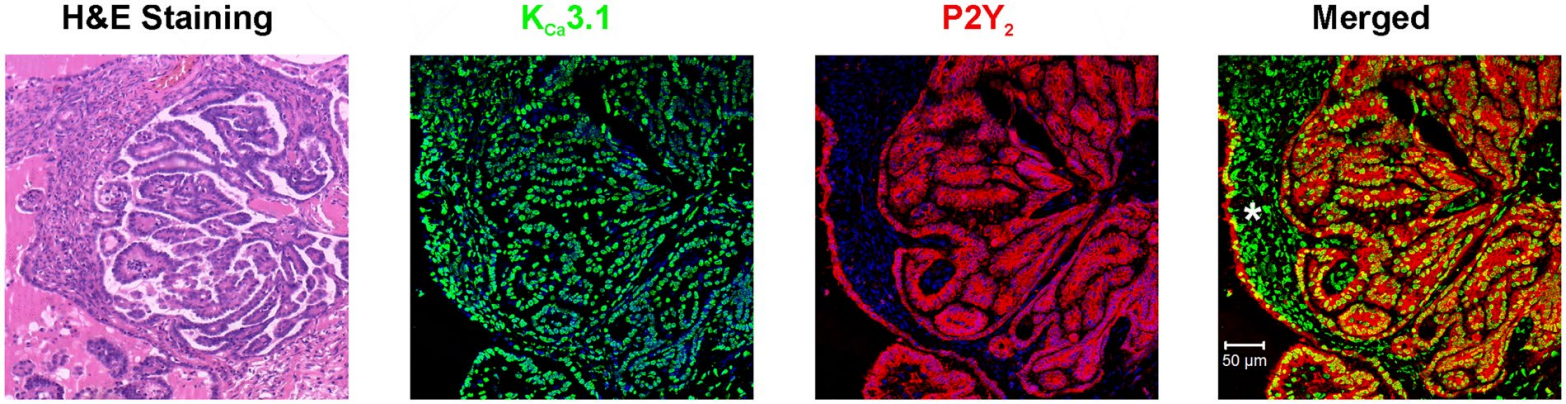
Co-localization of P2Y_2_ receptor and K_Ca_3.1 channel in human ovarian carcinoma. Expression of P2Y_2_ receptors and K_Ca_3.1 channels in slices from human ovarian carcinoma evaluated immunohistochemically. Biopsies from ovarian carcinoma tissue were collected, processed, and stained with hematoxylin-eosin and/or labeled with K_Ca_3.1- and P2Y_2_-specific antibodies that were revealed by second antibodies coupled to green and red fluorescent dyes, respectively. Nuclei in blue labeled with DAPI. Positive co-expression was detected in ovarian neoplastic cells (see merged image), whereas no signal was observed in control assays in which the primary antibody was omitted. Expression of K_Ca_3.1 was also observed in stromal cells that did not show neoplastic morphology (*); however, P2Y_2_ did not co-localize with K_Ca_3.1 in these cells. Patient 1, IC16-532-6.

It was also of importance to explore whether or not the main protein elements involved in the generation of the electrical and migration responses were expressed in tumor samples from human ovarian tumors. Figure 8 illustrates that K_Ca_3.1 and P2Y_2_ were expressed in biopsies of papillary serous carcinoma tumors (Patient 1, IC16-532-6) with a high degree of hypertrophy and hyperplasia. It is evident that human tissue expressed abundant K_Ca_3.1 channel protein with preferential localization in neoplastic cells; nevertheless, some other structures were labeled as well, mainly stromal cells without apparent neoplastic phenotype. The P2Y_2_ receptor signal was also localized primarily in areas where tumor cells presented K_Ca_3.1 protein expression, and several regions showed clear co-localization. Similar results were obtained in biopsies from 5 more patients (Patients numbers IC16-4831-1, IC11-738, IC11-7619-3, IC16-1288, and IC16-1050-9; see Supplementary Figure S3).

## Discussion

In this study we provide evidence that activation of specific receptors for ATP or histamine elicited an electrical response in SKOV-3 cells, a well-established cell model for human ovarian adenocarcinoma, with epithelial-like morphology. The electrical response was generated, most probably, as a consequence of the [Ca^2+^]_i_ increase generated by its release from internal stores, through the activation of specific receptors coupled to G proteins^16^. This idea is supported first, because a [Ca^2+^]_i_ increase was readily observed when the cells were stimulated by ATP or histamine at µM concentrations, as reported in previous studies, and also because the main electrical response corresponded to the generation of a Ca^2+^-dependent ionic current. The SKOV-3 response elicited by ATP and histamine had multiple phases; however, an outward current component was the most consistent and prominent. This response was mimicked by applying several purinergic agonists that, together, indicated participation of P2Y receptors sensitive to UTP. Nevertheless, comparatively small and fast inward responses elicited by ATP, but not by UTP (or ADP), seemed to indicate involvement of P2X channels in this particular response. The main UTP-sensitive P2Y receptors are the P2Y_2_, P2Y_4_ and P2Y_6_ subtypes, and expression analysis by PCR and immunocytochemistry showed that P2Y_2_ and P2Y_4_ were the main receptors expressed in SKOV-3 cells. However, given that the human P2Y_4_ receptor is not activated by ATP^22^, the potency sequence for the distinct agonists supported a main role for P2Y_2_ receptor involvement. For example, the P2Y_4_ receptors expressed in humans are also highly sensitive to Ap4A, but in SKOV-3 cells this agonist was much less effective than ATP or UTP; moreover, pharmacological data excluded involvement of P2Y_6_ since UDP was a weak agonist of the response^20^. Thus, nearly equipotent activation by ATP, UTP, and UTPγS, very low sensitivity to UDP, together with middle potency for Ap4A or ADP clearly indicated a main contribution of the P2Y_2_ subtype in the electrical responses elicited by purinergic agonists in SKOV-3 cells. This result also agrees with the EC_50_ of 400 nM for ATP found here for the outward response generated, since the range reported for the half-maximal effective concentration for P2Y_2_ ranged in different cell systems from 100 to 500 nM^33, 34^. The P2Y_2_ antagonist, AC-R118925, strongly inhibited the response generated by ATP or UTP, while other antagonistic drugs with high specificity for P2Y1 or the P2Y11 subtypes had no effect on SKOV-3 response. It is also important to emphasize the lack of effect by transmitters such as adenosine, acetylcholine or dopamine, suggesting the specificity of the SKOV-3 responses to ATP and histamine. This result is compatible with studies demonstrating that SKOV-3 cells do not present either expression of muscarinic receptors or a Ca^2+^ increase elicited by carbacholine, a muscarinic agonist^35^.

The outward-directed current response was carried mainly by K^+^ ions, as demonstrated by monitoring its reversal potential, which was close to the potential estimated for K^+^ ions in the recording conditions of −96.5 mV, as well as by changing the extracellular K^+^ concentration, which shifted the reversal potential of the response as predicted by the Nernst equation. Moreover, outward current responses elicited by ATP were potently blocked by TEA^+^ or 4-AP, two unspecific K^+^ channel blockers. Treatment of cells with more specific drugs indicated that the main pathway involved did not correspond to activation of two-pore K^+^ channels (TASK-3, TREK-1 and TREK-2), or to Ca^2+^-dependent K^+^ channels sensitive to apamin, some of which are proposed to be related to the cancer process in SKOV-3 cells^17, 18^. However, TRAM-34, a specific drug for Ca^2+^-dependent K^+^ channels of intermediate-conductance corresponding to the K_Ca_3.1 subtype, proved to be an effective blocker of the SKOV-3 response. Moreover, 1-EBIO, a specific positive modulator for this subtype of channels, showed an effect that was, as expected, dependent on the intracellular Ca^2+^ concentration. Thus, the mechanism that generates the ATP response in SKOV-3 cells more likely involves the activation of P2Y_2_ receptors, which produces a [Ca^2+^]_i_ increase and the subsequent opening of Ca^2+^-dependent K^+^ channels sensitive to TRAM-34 and 1-EBIO; these K^+^ channels are of the K_Ca_3.1 subtype expressed in SKOV-3 cells, as confirmed by Western blot analysis and immunofluorescence. Also, apparently K_Ca_3.1 channels were primed by P2Y_2_ activation, facilitating their opening. This regulatory mechanism was not studied further here; however, it has been shown that K_Ca_3.1 is post-translationally modulated by phosphorylation of a histidine residue in the C-terminal through nucleoside diphosphate kinase B^36^. A similar mechanism might be responsible for the effect observed during ATP-elicited response in SKOV-3 cells.

All functional and pharmacological evidence was also supported by results of specific downregulation of P2Y_2_ or K_Ca_3.1 protein expression using the esiRNA-transfection method. Silencing P2Y_2_ or K_Ca_3.1 gene produced a strong downregulation of the respective protein expression, which had a strong impact on generation of electrical response elicited by ATP or UTP that was eliminated. Moreover, a significant reduction in [Ca^2+^]_i_ increase elicited by these agonists was observed using fluorometric analysis. In average, reduction in [Ca^2+^]_i_ increase in cells transfected with P2Y_2_-esiRNA was not as robust as that observed for the electrical response, indicating that [Ca^2+^]_i_ increase was probably insufficient for K_Ca_3.1 channel activation, and also probably to deficiencies in the P2Y_2_ receptor mechanism that primed the channels. Another observation was that K_Ca_3.1 esiRNA-transfected cells also showed a reduction in [Ca^2+^]_i_ response when ATP or UTP was applied. This might indicate a role of K^+^ channels in [Ca^2+^]_i_ increase, probably through membrane potential regulation that would affect voltage-dependent mechanisms and/or reduce Ca^2+^ influx, among several other possibilities. Similarly, it has been reported that downregulation of nucleoside diphosphate kinase B expression reduces K_Ca_3.1 channel activity in CD4 T cells and decreases Ca^2+^ influx^36^.

Furthermore, E_rev_ obtained during the development of the inward current component seems to indicate the flux of Cl^−^ ions. However, in preliminary results a specific blocker for Ca^2+^-dependent Cl^−^ channels of the TMEM16-A subtype did not inhibit this component; hence, further studies are necessary to define its nature and molecular identity. Finally, a similar current response was observed in the ovarian carcinoma cell line CaOV-3.

A relationship between K_Ca_3.1 channel activation and the process of cell migration has been documented in both normal and pathological conditions^29^. Therefore, we have tested whether or not opening K_Ca_3.1 channels with ATP was an effective way to increase SKOV-3 migration at the low ATP concentrations that activated the electrical response. The results showed that 3 µM ATP application promoted SKOV-3 cell migration and that this was also blocked by TRAM-34 and mimicked by 1-EBIO. A strong inhibition of cell migration was also achieved by downregulation of either P2Y_2_ or K_Ca_3.1 protein, an effect that was observed on motility promoted by either ATP, UTP or 1-EBIO. This strongly suggests that an increase of extracellular ATP in the low µ-molar range would have an important consequence in the tumor microenvironment; an effect that would be influenced by a concomitant increase in histamine, which might act directly or indirectly on specific receptors (e.g., by promoting the release of ATP as shown in other cell systems)^37^.

Together, the results presented here support the idea that an increase in ATP concentration within the tumor microenvironment might alter the function of channels such as K_Ca_3.1. This would promote cell motility, an effect possibly potentiated by several factors including an increase in the expression of the molecules involved, specifically P2Y_2_ receptors and K_Ca_3.1 channels in neoplastic cells. In fact, in distinct cancer cell types it has been shown that cell motility in general is highly dependent on [Ca^2+^]_i_, acting through a complex machinery of molecules which are, in most cases, Ca^2+^-dependent^29^. Among the molecules involved, those that allow the flux of ions through the cell membrane are essential for the process. Both Ca^2+^-dependent K^+^ channel and Cl^-^ channel activation are required for cell motility, allowing the needed movement of water across the membrane and the cell volume changes, which are membrane mechanisms that are also active during the process of metastasis^10, 38^. There is evidence indicating that this might be the case in some cancer cell types specifically regarding K_Ca_3.1 channels; for example, brandykinin activation of K_Ca_3.1 channels in human glioma cells promotes their migration in both *in vitro* and *in vivo* models^31, 39^. In addition, elevated levels of K_Ca_3.1 expression in breast cancer cells^9^ and cell renal carcinoma^40^ correlate with tumor grade and metastatic status. Here, we also show that molecules involved in the SKOV-3 electrical response were robustly expressed in biopsies from human ovarian tumors. A strong expression of both P2Y_2_ receptors and K_Ca_3.1 channels was observed in human ovarian tumors, and they specifically co-localized in neoplastic cells. Thus, it is proposed that opening of K_Ca_3.1 channels by ATP (and histamine) in human ovarian cancer cells might be one of the mechanisms deregulated during the cancer process, possibly explaining the effects that this transmitter has during tumorigenesis.

It is well known that K_Ca_3.1 channels play a main role in the migration of microglial cells, as part of the normal immune response in the nervous system^30^, as well as in human dendritic cells^41^ and in activated human T cells^42^. In this context, there are physiological conditions in which ovarian cells might require the capacity to migrate; for example, during ovulation the ovarian superficial epithelium suffers a rupture that allows oocyte release, which occurs through a mechanism similar to an acute inflammatory reaction^43^. After the gamete is released the ovarian surface wound is repaired by the epithelium. The latter process requires cell migration, as indicated by the profile of genes expressed during ovulation, that shows a strong correlation with important epithelial functions, such as the inflammation reaction, angiogenesis, extracellular matrix remodeling and cell-to-cell contact^44^. Both ATP and histamine signaling have been proposed to be involved in ovulation^15^; in this manner, transmitter-activated membrane ionic currents in the ovarian superficial epithelium might participate during this specific physiological condition, a hypothesis that requires further studies. Nevertheless, an important role for K_Ca_3.1 in general epithelial secretory activity is expected, as suggested by its broad expression in most of the epithelial cells analyzed^45^.

In summary, in this study we show that purinergic and histaminergic stimulation generated electrical responses in SKOV-3 cells through the opening of membrane ion channels. This has two important implications: first, two main transmitters that are commonly found in increased concentrations in the tumoral microenvironment exerted direct actions on membrane conductivity pathways; and second, activation of one of these pathways, the K_Ca_3.1 channel subtype, was directly involved in SKOV-3 cell motility, an important phenomenon in cancer. Since the P2Y_2_ receptor and the K_Ca_3.1 channel are co-expressed in neoplastic cells from human ovary, we propose that they may be useful tumor markers as well as targets for therapy to halt ovarian cancer progression.

## Methods

### Cell cultures

The human ovarian cancer cell line SKOV-3 was purchased from American Type Culture Collection (ATCC; Manassas, VA, USA). Cells were cultured at 37 °C in a humidified atmosphere containing 95% air: 5% CO_2_ in RPMI1640 growth medium with L-glutamine (Mediatech, Manassas, VA, USA), supplemented with 10% fetal bovine serum (FBS; Gibco, Waltham, MA, USA) and 1% antibiotic-antimicotic mix (streptomycin, penicillin and amphotericin B; Life Technologies, USA). SKOV-3 cells were plated onto 12-mm-diameter coverslips in 12-well culture dishes and after 48 h in culture were used for electrical recording or other experimental protocols such as migration assays or specific protein detection by Western blot or immunolabeling. In some experiments, human ovarian adenocarcinoma CaOV3 cells obtained from ATCC were cultured in a similar manner for electrophysiological recording. As control cells, cortical neurons were cultured from brain cerebral cortex of E18 Sprague-Dawley rat embryos^46^. Briefly, neurons were dissociated enzymatically and mechanically, seeded on poly-D-lysine-coated coverslips and maintained in neurobasal medium supplemented with 10% FBS. Cultures were processed for immunocytochemistry after 7 days *in vitro*.

### Electrophysiology

Whole-cell recordings were performed at room temperature (23–26 °C) using the Axon 200B patch-clamp amplifier (Axon Instruments; Sunnyvale, CA, USA). Currents were regularly recorded at a holding membrane potential of −40 mV, digitized and stored using the A/D converter Digidata 1400 and pClamp 10 software (Axon Instruments) for subsequent analysis. The extracellular bath solution adjusted to pH 7.4 contained the following (in mM): 140 NaCl, 3 KCl, 1 CaCl_2_, 1 MgCl_2_, and 10 HEPES. Patch-clamp pipettes (3–5 MΩ) were filled with internal solution adjusted to pH 7.4 containing (in mM): 130 KCl, 5 NaCl, 2 EGTA, 1 MgCl_2_, 10 HEPES, 2 Mg-ATP, and 0.2 Na-GTP. The estimated Ca^2+^ concentration in this solution was 10 nM. In some experiments, the estimated free Ca^2+^ concentration was increased to 300 nM by changing the EGTA concentration to 2 mM and adding 1.5 mM CaCl_2_. External solutions with various K^+^ concentrations were prepared by equimolar substitution of NaCl by KCl to 10, 30 or 60 mM or the KCl concentration was decreased to 1 mM without compensation. Agonists and other drugs were added to the external solution from stock solutions to reach the desired dilution and applied through superfusion. In most cases, peak currents generated at -40 mV by drug superfusion were used in the analysis. Current-voltage (I/V) relationships were built by changing the membrane potential from −120 to + 60 mV in 20-mV steps (150 ms) while the cells were held at −40 mV, and the peak membrane current values at the beginning (20 ms) of each step were plotted as in Fig. 4. In some other cases (Fig. 2B) the membrane potential was held at a desired value while drugs were superfused.

### Fluorometry

Intracellular [Ca^2+^] was monitored using fluorometric techniques. SKOV-3 cells were grown on coverslips and loaded with Fluo4-AM (5 mM; Molecular Probes, Eugene, OR, USA) in the external solution for 30 min at 37 °C. Then, cells were washed with external solution for 10 min to eliminate excess dye and placed in a constant-flow recording chamber that allowed visualization of the cells using an inverted fluorescence microscope (Olympus; Melville, NY, USA). Drugs were applied by superfusion, and responses were recorded with an Evolution QEi camera (Media Cybernetics; Bethesda, MD, USA). Sequences of images were analyzed using the Image-Pro Plus software (Media Cybernetics) and ImageJ software (NIH; Bethesda, MD, USA).

### Reverse Transcription Polymerase Chain Reaction

Total RNA from SKOV-3 cells was purified using the guanidine isothiocyanate method. First strand cDNA was synthesized using 2 μg of DNase-treated RNA as template, 1 µg of oligo(dT), 1.5 µg of random hexamers, and reverse transcriptase. The cDNA was used as template in a polymerase chain reaction to amplify cDNA fragments for *β-actin*, P2Y_2_, P2Y_4_, and P2Y_6_ transcripts. All the PCR programs started at 96 °C for 2 min and finished at 72 °C for 5 min. The amplification cycles consisted in 40 s at 96 °C, 40 s at the specific annealing temperature for each primer set, and 40 s at 72 °C.

The sequences of the oligonucleotides, the annealing temperatures, and the number of PCR cycles used were as follows: P2Y_2_r, forward GGACGAACTGGGATACAAGTGT, reverse GTGGACTCTGTCCGTCTTGAGT, annealing temperature 55 °C, 30 cycles; P2Y_4_r, forward GGGACTAACTGCAGGCAGAG, reverse GATACACATCAGGCCCGTCT, annealing temperature 60 °C, 40 cycles; P2Y_6_r, forward TTTCAAGCGACTGCTGCTAA, reverse TGGCATAGAAGAGGAAGCGT, annealing temperature 55 °C, 30 cycles; and *β-actin*, forward GGGTCAGAAGGATTCCTATG, reverse GGTCTCAAACATGATCTGGG, annealing temperature 55 °C, 25 cycles. The amplified products were gel isolated and subcloned into the pJET1.2/blunt vector (Thermo Fisher Scientific, Waltham, MA USA); then, the nucleotide sequences were confirmed by automatic sequencing.

### Western blot

Cultured SKOV-3 cells were scraped in Laemmli buffer and boiled for 5 min. For electrophoresis, samples were fractionated in a 10% SDS-polyacrylamide gel and transferred to a nitrocellulose membrane (BioRad; Hercules, CA, USA). Membranes were blocked for 1 h at room temperature in 150 mM NaCl, 20 mM Tris, pH 7.4, and 0.1% Tween 20 (TBS-T) containing 5% nonfat dry milk and then incubated overnight at 4 °C with the appropriate mouse monoclonal antibody (1:1000) directed against the K_Ca_3.1 channel protein (ALM-051, Alomone; Jerusalen, Israel). After washing with TBS-T, membranes were incubated for 1 h at 37 °C with HRP-conjugated goat anti-rabbit antibody (Zymed; Grand Island, NY, USA) in TBS-T. The immunoreactive proteins were detected by chemiluminescence, and images were analyzed with ImageJ Software.

### Immunocytochemistry

For P2Y_2_, P2Y_4_ or K_Ca_3.1 channel immunostaining, cells were treated with the corresponding antibody (anti-P2Y_2_ (1/100) APR010; anti-P2Y_4_ (1/100) APR006; or anti-K_Ca_3.1 (1:1000) ALM051; all from Alomone, Jerusalem, Israel). In all cases the cells were fixed in 4% paraformaldehyde in PBS for 20 min at room temperature. The fixed cultures were permeabilized with 0.1% Tween-20, blocked with 5% goat serum in PBS for 30 min and incubated overnight at 4 °C with the antibodies diluted in PBS containing 5% goat serum and 0.1% Tween-20. Then, cells were rinsed and incubated for 2 h at room temperature with 1:100 anti-mouse IgM-G conjugated with Alexa 488 (Molecular Probes). After three washes with PBS, samples were stained with 4′,6-diamidino-2-phenyl-indole dihydrochloride (DAPI 14 mM, from Molecular Probes). Finally, the samples were mounted on VectaShield (Vector Laboratories; Burlingame, CA, USA), and the preparations were visualized under a laser scanning confocal microscope LSM510 (Zeiss; Oberkochen, Germany).

### Migration assay

Transwell assays were performed using 12-well plates containing 8-mm polyethylene hanging cell culture inserts (Millipore; Billerica, MA, USA). The cells were seeded at the apical side of the chamber. The basolateral side was filled with medium supplemented with drugs according to the particular experimental conditions. After 16 h of incubation with drugs, cells attached on the lower face of the inserts were fixed with 4% paraformaldehyde in PBS for 10 min and stained with 10 µg/ml propidium iodide. The samples were visualized under a microscope, pictures were taken and images were analyzed using ImageJ software (NIH) for quantification.

### Cell proliferation assay

To analyze cell proliferation and viability, mitochondrial activity of the whole culture was assessed by using the 3-(4,5-dimethylthiazol-2-yl)-5- (3-carboxymethoxyphenyl)-2 (4-sulfophenyl)-2H-tetrazolium salt (MTS) assay (Promega; Wisconsin, USA). For this, cells were cultured in 96-well plates in RPMI-1640–10% FBS, and after 24 h they were transferred to serum-free RPMI-1640 for 8 h. After this, an appropriate stimulus was applied, and the cultures were incubated for an additional 16 h. Finally, the MTS assay was performed as described by the manufacturer. Results were quantified as fold increase of absorbance in experimental conditions relative to unstimulated cells.

### esiRNA transfection

SKOV-3 cells were plated at 4 × 10^4^ cells per well in 24-well plates and allowed to attach overnight. Endonuclease prepared small interfering RNAs (esiRNAs) were commercially synthesized (Sigma-Aldrich, St. Louis MO, USA) using either 420 bp length of P2Y_2_ receptor (EHU156731; P2Y_2_-esiRNA) or 472 bp length of K_Ca_3.1 channel gene (EHU035251; K_Ca_3.1-esiRNA), targeting human sequences NM_002564 and NM_002250, respectively. Then, for P2Y_2_ receptor or K_Ca_3.1 channel knockdown, cells were transfected with 150 ng per well of P2Y_2_-esiRNA or K_Ca_3.1-esiRNA, respectively, by using Lipofectamine 3000 (Invitrogen, Grand Island NY, USA) to deliver esiRNA into the cells following the method indicated by the manufacturer. As a non-targeting control (referred to as CNT), was transfected in the same condition the esiRNA of enhanced green fluorescent protein (EHUEGFP, Sigma-Aldrich), all responses of transfected SKOV-3 cells with either P2Y_2_-esiRNA or K_Ca_3.1-esiRNA were compared versus those of the CNT group. Twenty-four to 72 h after transfection, esiRNA-treated SKOV-3 cells maintained in culture were recorded electrically, or used for immunocytochemistry or fluorometric assays, as well as for migration quantification using the methods described above.

### Immunohistochemistry in human ovarian cancer biopsies

To explore whether human ovarian tumors express P2Y_2_ receptor and K_Ca_3.1 channel proteins, biopsies of patients with ovarian carcinoma were analyzed by immunohistochemistry. The samples were obtained from the Instituto Nacional de Cancerología (INCAN) México, where clinical histories of all patients are archived, in accordance with ethical procedures approved by the Bioethics Committee. The samples used in the present study correspond to 6 patients diagnosed with ovarian carcinoma. The characteristics of the carcinomas analyzed are as follows: Patient 1 (IC16-532-6), 44 years of age diagnosed with low-grade papillary serous carcinoma; Patient 2 (IC16-4831-1), 61 years of age diagnosed with high-grade papillary serous carcinoma; Patient 3 (IC11-7381), 56 years of age with endometrioid G3-type carcinoma; Patient 4 (IC11-7619-3), 70 years of age diagnosed with high-grade serous carcinoma; Patient 5 (IC16-1288), 37 years of age with endometrioid G2-type carcinoma; and Patient 6 (IC16-1050-9), 57 years of age diagnosed with high-grade papillary serous carcinoma. Paraffin-embedded human ovarian biopsies were cut at 10-µm intervals and the slices attached to gelatinized slides. Paraffin was eliminated by incubating with xylene, and slices were rehydrated by passing them through a series of five concentrations of ethanol (from 100% to 50%) and washed with PBS. Antigens were exposed by incubating in 10 mM sodium citrate, pH 6, for 15 min and then equilibrated in PBS. For immunohistochemistry, samples were incubated overnight with the appropriate antibodies (1:80 anti-K_Ca_3.1, and 1:100 anti-P2Y_2_, both from Alomone, Jerusalem, Israel), and non-specific sites were blocked with 3% BSA in PBS. The next day, samples were washed with PBS and incubated for 1 h at room temperature with anti-mouse Alexa Fluor 488 (1:100; Jackson ImmunoResearch, West Grove, PA, USA) or anti-rabbit Cy5 (1:100; Life Technologies, Carlsbad, CA, USA). After three washes with PBS, samples were stained with 1:1000 DAPI (Molecular Probes). Finally, the samples were mounted on VectaShield and analyzed by confocal microscopy.

Tissue samples were also stained with the hematoxylin and eosin technique, and sections were visualized and analyzed under a microscope; representative images were acquired with a Leica ICC50 HD (Leica Microsystems, Wetzlar, Germany).

### Reagents

All the following drugs were purchased from Sigma-Aldrich (St. Louis, MO, USA) or Tocris Bioscience (Bristol, UK): Adenosine 5′-triphosphate (ATP); uridine 5′-triphosphate (UTP); histamine; adenosine 5′-(γ-thio)-triphosphate (ATPγS); P^1^,P^4^-Di(adenosine-5′) tetraphosphate (Ap4A); 2-(Methylthio) adenosine 5′-triphosphate (2-MeSATP); 2′(3′)-O-(4-Benzoylbenzoyl)adenosine 5′-triphosphate tri(triethylammonium) salt (Bz-ATP); 5-Bromo-2′-deoxyuridine (5Br-UDP); adenosine 5′-diphosphate (ADP); 2-(Methylthio) adenosine 5′-diphosphate (2-MeSADP); Uridine 5′-diphosphate (UDP); dopamine; adenosine; acetylcholine; 5-[[5-(2,8-Dimethyl-5*H*-dibenzo[*a*,*d*]cyclohepten-5-yl)-3,4-dihydro-2-oxo-4-thioxo-1(2*H*)-pyrimidinyl]methyl]-*N*-2*H*-tetrazol-5-yl-2-furancarboxamide (AR-C118925); 4,4′-(Carbonylbis(imino-3,1-(4-methyl-phenylene)carbonylimino)) bis(naphthalene-2,6-disulfonic acid) tetrasodium salt (NF340); 2′-Deoxy-*N* ^6^-methyladenosine 3′,5′-bisphosphate tetrasodium salt (MRS2179); (E,E)-1,7-bis(4-Hydroxy-3-methoxyphenyl)-1,6-heptadiene-3,5-dione (curcumin); R(+)-Arachidonyl-1′-hydroxy-2′-propylamide (R(+)-methanandamide); tetrapentylammonium chloride (TPenA); L-2-Amino-4-(methylthio)butanoic acid (L-Methionine); tetraethylammonium chloride (TEA^+^); 4-Aminopyridine (4-AP); 1-[(2-Chlorophenyl)diphenylmethyl]-1H-pyrazole (TRAM-34); 1-Ethyl-1,3-dihydro-2H-benzimidazol-2-one (1-EBIO); Ethylene glycol-bis (2-aminoethylether)-N,N,N′,N′-tetraacetic acid (EGTA). Uridine 5′-(γ-thio)-triphosphate (UTPγS); 2-[(5-Ethyl-1,6-dihydro-4-methyl-6-oxo-2-pyrimidinyl)thio]-N-[4-(4-methoxyphenyl)-2-thiazolyl] acetamide (T16Ainh-A01); and apamin. All other common salts were from Sigma-Aldrich or J.T. Baker (Phillipsburg, NY, USA).

### Statistical Analysis

AII data are expressed as mean ± S.E.M. The means of two groups were compared using a Student’s t-test, or when appropriate, by analysis of variance followed by post-hoc comparisons of individual means using the Bonferroni correction. Statistical analysis was performed using GraphPad Prism software. Differences were considered to be significant at P < 0.05.

## Electronic supplementary material

Supplementary Figures
