# Supplementary material for: Kca3.1 Activation Via P2y2 Purinergic Receptors Promotes Human Ovarian Cancer Cell (Skov-3) Migration
Source: Sci Rep. 2017 Jun 28;7:4340. doi: 10.1038/s41598-017-04292-6 (PMC5489490; doi:10.1038/s41598-017-04292-6)
Supplement: Supplementary file 1 — Supplementary Figures [file 41598_2017_4292_MOESM1_ESM.pdf]

**$K_{Ca}$3.1 ACTIVATION VIA $P2Y_2$ PURINERGIC RECEPTORS PROMOTES HUMAN OVARIAN CANCER CELL (SKOV-3) MIGRATION**

By

Robles-Martínez L., Garay E., Martel-Gallegos M.G., Cisneros-Mejorado A., Pérez-Montiel D., Lara A. & Arellano R.O.

Supplementary Figure 1 (S1)

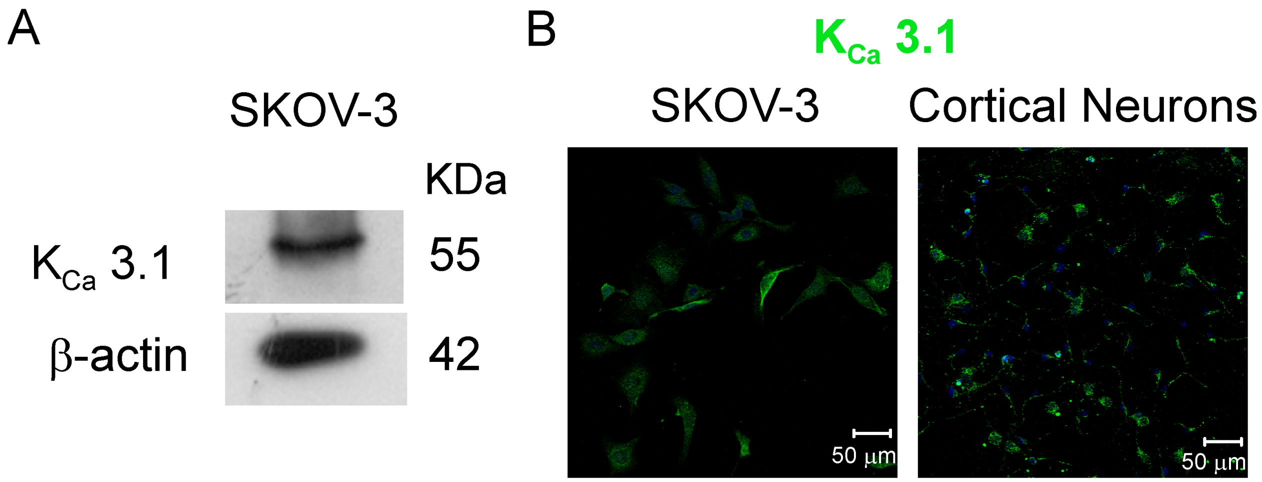


**Figure S1. KCa3.1 channel protein expression in SKOV-3 cells.**

**A**. $K_{Ca}$3.1 was detected in protein homogenates of SKOV-3 cells as a band of 55 KDa by Western blot; β-actin was detected in the same samples as control. **B**. The same antibody against $K_{Ca}$3.1 in **A** was used to detect the protein by immunofluorescence in SKOV-3 cells, and as positive control, in cortical neurons maintained in culture.

Supplementary Figure 2 (S2)

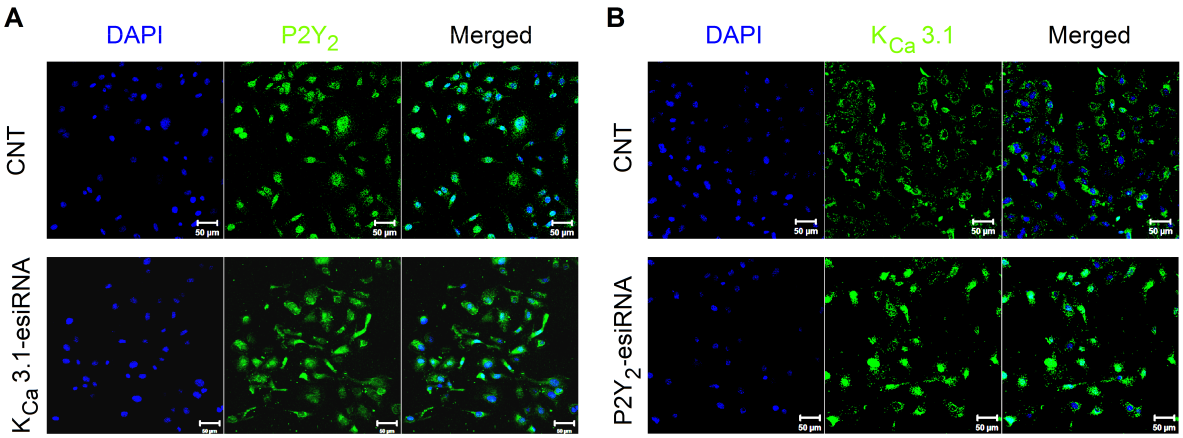


**Figure S2. $P2Y_2$ and $K_{Ca}3.1$ protein expression in esiRNA transfected SKOV-3 cells.**

**A.** Analysis by immunocytochemistry after 48 h of esiRNA treatment in control (CNT) and $K_{Ca}3.1$-esiRNA-treated groups. Panels show the fluorescence signal for DAPI (blue) in the first column, the signal obtained with a specific antibody against $P2Y_2$ receptor protein (in green) in the second column, and the corresponding merged image. **B.** Similar analysis was made in $P2Y_2$-esiRNA-treated cells using an antibody against $K_{Ca}3.1$ channel protein.

Supplementary Figure 3 (S3)

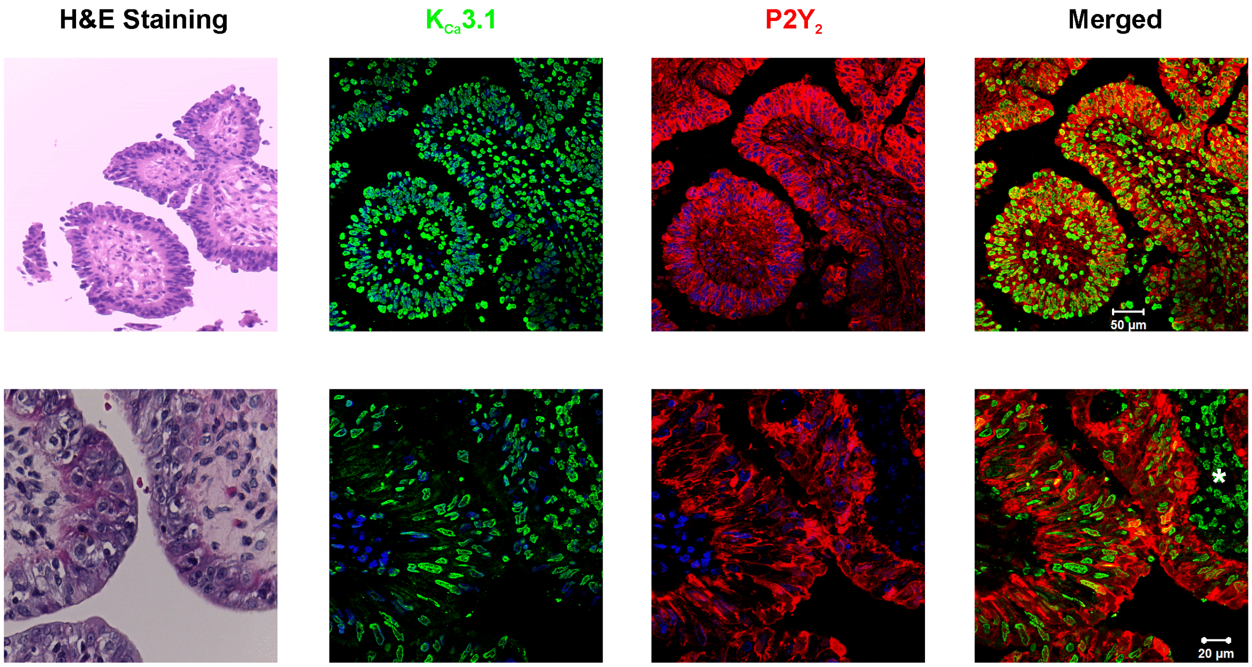


**Figure S3. Co-localization of $P2Y_2$ receptor and $K_{Ca}3.1$ channel in human ovarian carcinoma.**

Expression of $P2Y_2$ receptors and $K_{Ca}3.1$ channels in slices from human ovarian carcinoma evaluated immunohistochemically as in Figure 8. Patient 2 (IC16-4831-1) diagnosed with high-grade papillary serous carcinoma (first row), and Patient 3 (IC11-7381) with endometrioid G3-type carcinoma, respectively.
